# Phosphatidylserine-targeting antibody induces M1 macrophage polarization, promotes myeloid derived suppressor cell differentiation and boosts tumor-specific immunity

**DOI:** 10.1186/2051-1426-1-S1-P154

**Published:** 2013-11-07

**Authors:** Xianming Huang, Yi Yin, Dan Ye, Rolf Brekken, Philip Thorpe

**Affiliations:** 1 University of Texas Southwestern Medical Center, Dallas, TX, USA

## 

Phosphatidylserine (PS) is a potent immunosuppressive lipid typically segregated to the inner leaflet of the plasma membrane. PS is externalized on tumor vasculature, tumor-derived exosomes, and tumor cells in the tumor microenvironment and externalization is markedly enhanced by therapy (e.g., radiation, chemotherapy, and/or androgen deprivation). Externalized PS interacts with immune cells where it actively promotes immunosuppression and tumor progression by expansion of myeloid derived suppressor cells (MDSCs) and M2-like tumor associated macrophages (TAMs). Bavituximab is a PS-targeting antibody that is being evaluated in multiple late-stage clinical trials in cancer patients. Here we show that treatment of PC3 tumor-bearing mice with 2aG4, a murine-version of bavituximab, significantly depleted M2-likeTAMs and MDSCs and increased the presence of M1-like TAMs and mature dendritic cells. In addition, PS blockade markedly altered the cytokine balance in the tumor microenvironment from immunosuppressive to immunostimulatory. Furthermore, in the immune-competent TRAMP mouse prostate tumor model, combination treatment of anti-PS antibody with castration induced strong tumor-specific T-cell immunity that resulted in significantly improved tumor free long-term survival and apparent cures in 35% of the animals, compared to no long-term survivors in groups treated with either single agent. In vitro studies confirmed that anti-PS re-polarized TAMs from an M2 to M1-like phenotype and drove MDSCs to differentiate into M1-like macrophages and functional dendritic cells. These data suggest that PS expression on the external cell surface defines an upstream immune checkpoint that is primarily responsible for expansion of MDSCs and M2-like TAMs in tumors. The results further support that blockade of the PS signal by bavituximab treatment can reverse this immune checkpoint suppression and promote therapeutically effective anti-tumor immunity.

